# A Brief Review on Aflatoxicosis in Aquaculture With a Focus on Fish

**DOI:** 10.1155/anu/3130230

**Published:** 2024-12-14

**Authors:** Mina Ziarati, Ahmad Imani, Hamed Ghafarifarsani, Deepa Bhatt

**Affiliations:** ^1^Department of Microbiology, National Center for Survey and Disease Diagnosis, Iranian Veterinary Organization (IVO), Bushehr, Iran; ^2^Department of Fisheries, Faculty of Natural Resources, Urmia University, Urmia, Iran; ^3^Department of Animal Science, Chaharmahal and Bakhtiari Agricultural and Natural Resources Research and Education Center, Agricultural Research, Education and Extension Organization (AREEO), Shahrekord, Iran; ^4^Department of Aquaculture, College of Fisheries, Guru Angad Dev Veterinary and Animal Sciences University, Ludhiana, Punjab, India

**Keywords:** aflatoxicosis, aquaculture, aquafeed, feed contaminants, fungal toxins, mycotoxins

## Abstract

Feed quality is among the most determinative criteria for aquaculture success. Along with feed ingredient quality and its production process, feed storage conditions would also affect feed quality, especially in terms of adventitious toxins. Mycotoxins are frequent food and feed contaminants and are considered important health threats to both human and animal health. In this context, the effects of mycotoxins on aquatic animals were reviewed with an emphasis on aflatoxin B_1_ (AFB_1_), which is obviously reported in aquafeed. Severe tissue damage, increased susceptibility to infectious diseases, compromised immune system function, and increasing unknown death risks are among the most frequent symptoms of aflatoxicosis in aquatic animals. The lowest observable effect level for AFB_1_ has also been documented for different fish species. Considering the importance of such fungal toxins on the economic viability of aquaculture enterprises, it is recommended that further knowledge be obtained concerning the safe levels of AFB_1_ in terms of fish health and final product safety to human consumers.

## 1. Introduction

World aquaculture production is projected to increase by 62% (35 million tons) from 2010 to 2030, with over 90% of such growth occurring in lower middle-income countries [1]. Aquaculture has recently transformed into a more sustainable and productive industry model, mainly due to cost feed formulation strategy and search for fishmeal alternatives in the last decade [2, 3]. Fish feed is an essential part of the aquaculture industry and significantly contributes to fish production costs and quality [4]. It plays a pivotal role in determining the success and profitability of fish farming. Ensuring high-quality feed is vital for optimizing fish health and growth. However, it has been reported that the feed is prone to being contaminated by fungal toxins [5, 6]. The risk of toxin occurrence in feeds increases at temperatures above 27°C, humidity above 62%, and feed moisture content above 14%. Inappropriate feed storage is a common predisposing factor for fungal and mold growth [7] and poses severe health issues in terms of human health and animal production costs and health [8, 9]. Contamination with mycotoxins might result in decreased nutritional values of ingredients and finished feed [10]. Mycotoxins might be responsible for hepatocellular and neurologic injuries, hypoimmunity, cancer, and even an increased mortality rate [11]. The toxic effects of mycotoxins depend not only on their dietary contents but also on the duration of toxin exposure and fish species, gender, and ontogenic stage [12]. Although there have been some reports regarding decreasing toxin bioavailability using dietary additives, including yeast cell wall, clay minerals, and pro/post-biotics, mycotoxins are still among the main risks of reduced fish growth performance and immune competence [5, 13–17]. Moreover, the long-term ingestion of feeds with low levels of mycotoxins or acute exposure to high dietary contents might be a reason for the unexplained mortalities occasionally observed in fish farms [18–20]. While the effects of mycotoxins are relatively well-known in most terrestrial farm animals, the outcomes of dietary mycotoxin contamination on aquaculture species have yet to be extensively studied. Therefore, the present review focused on the effect of mycotoxins, especially aflatoxin B_1_ (AFB_1_), on aquatic animals in terms of fish growth performance, digestive tract physiology, immune system functionality, and intestinal barrier integrity. Such understanding plays a crucial role in managing the adverse effects caused by mycotoxins and increasing social awareness regarding the presence of such toxins in aquafeed.

## 2. Mycotoxins

Mycotoxins are low molecular weight secondary fungal metabolites (MW∼700 Da) produced by *Aspergillus*, *Penicillium*, *Fusarium*, and *Alternaria* species [21–23]. Fungi frequently contaminate agricultural commodities throughout the world [24]. Humans and animals are exposed to mycotoxins mainly through the alimentary tract; however, inhalation and skin contact might also be possible [25]. Environmental factors, especially warm climates and irregular precipitation due to climate change, naturally promote fungal growth and increase the risk of mycotoxin occurrence in agricultural products [26, 27].

**Figure 1:**
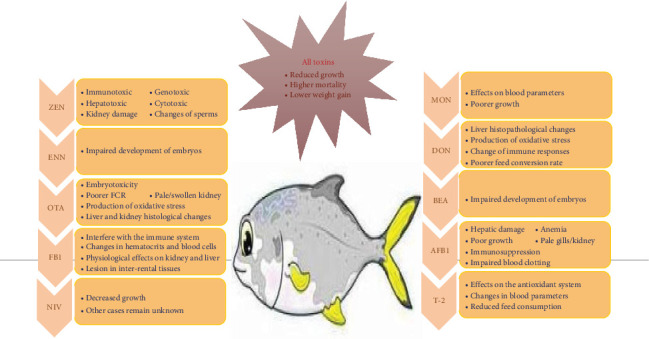
Chemical structure of the most common mycotoxins. AFB_1_, aflatoxin B_1_; BEA, beauvericin; DON, deoxynivalenol; ENN, enniatins; FB_1_, fumonisin B_1_; MON, moniliformin; NIV, nivalenol; OTA, ochratoxin A; T-2, T-2 toxin; ZEN, zearalenone.

**Figure 2:**
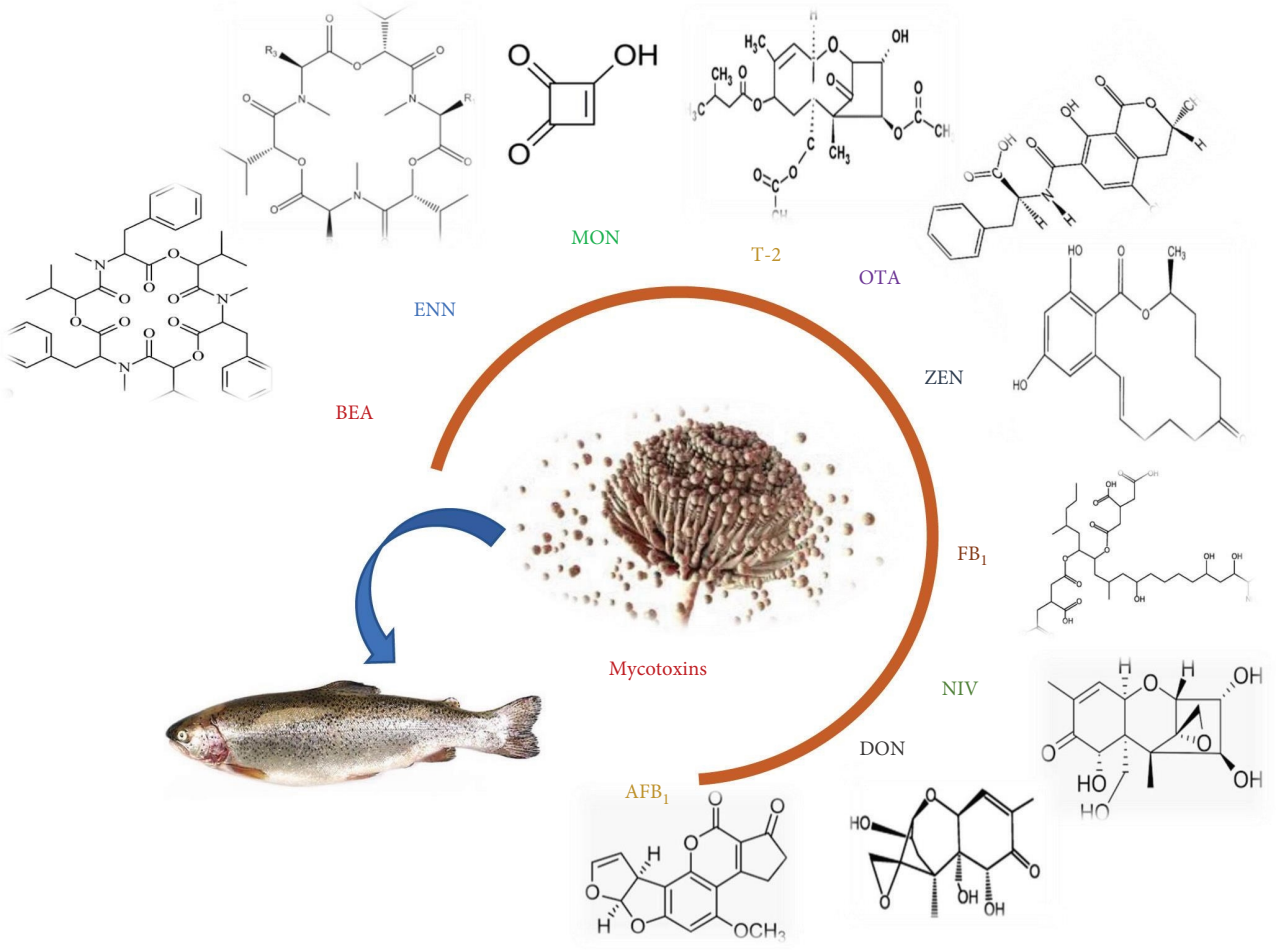
The effect of mycotoxins on fish. AFB_1_, aflatoxin B_1_; BEA, beauvericin; DON, deoxynivalenol; ENN, enniatins; FB_1_, fumonisin B_1_; MON, moniliformin; NIV, nivalenol; OTA, ochratoxin A; T-2, T-2 toxin; ZEN, zearalenone.

Mycotoxins come in various structural forms (Figure 1), from four simple-carbon compounds to complex substances [28]. More than 500 different mycotoxins have been isolated and chemically characterized according to previous data [29]. The 10 most common and hazardous feed mycotoxins include AFB_1_, deoxynivalenol (DON), nivalenol (NIV), zearalenone (ZEN), ochratoxin A (OTA), T-2 toxin (T-2), fumonisin B_1_ (FB_1_), moniliformin (MON), enniatins (ENN), and beauvericin (BEA) [30]. Among them, aflatoxins (AFs) are mostly studied as fungal toxins in aquaculture species and seem to affect industry development worldwide [31]. The effects of mycotoxins on fish are shown in Figure 2.

### 2.1. AFs

AFs are the first mycotoxins discovered after a case of what was later found to be acute aflatoxicosis, turkey “X” disease, that resulted in the death of around 100,000 turkeys in the 1960s [32]. They are the most studied and well-characterized mycotoxins. AFs are highly toxic, carcinogenic, teratogenic, and mutagenic secondary metabolites primarily produced by the conidial fungi of the genus *Aspergillus*, mainly *A. flavus* and *A. parasiticus* [9, 33, 34]. The potential teratogenic characteristics of AFs play a vital role in human and animal malignancies. It is worth mentioning that about 4.5 billion people worldwide are at risk of AF exposure, mainly in poor and undeveloped countries [11].

Feed AF contamination has been reported to result in decreased fish growth performance, anemia, hemorrhage, liver function impairment, higher vulnerability to infectious diseases, and increased mortality [7]. Clinical signs associated with aflatoxicosis in fish include pale gill coloration and pathological tissue changes, altered blood indices, and lower growth rates with subsequently decreased weight gain (WG), reduced survival rate, darkening/yellowing of the body, and abnormal behavior [35–37]. In addition, dietary AFs might affect the nutritional value of fish muscle tissues [38, 39].

**Figure 3:**
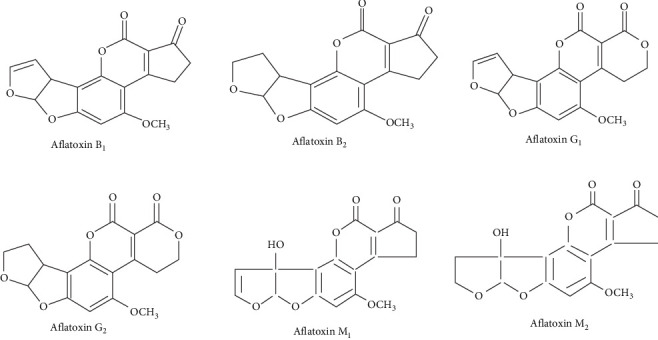
Chemical structure of the most important aflatoxins [41], with some modifications.

According to their natural blue or green fluorescence, AFs have several main types, including AFB_1_, AFG_1_, AFM_1_, AFB_2_, AFG_2_, and AFM_2_ [30, 40], which are illustrated in Figure 3. They are common in feed ingredients, finished feeds, and aquatic environments [42]. Among different AFs, AFB_1_ is considered the most common natural carcinogenic compound by the United States Food and Drug Administration. Its hepatotoxic effects, mutagenicity, carcinogenicity, teratogenicity, and immune system suppression have been confirmed in fish species [14, 31]. Meanwhile, AFM_1_ does not seem to be an important threat to fish health [30].

#### 2.1.1. AFB_1_

The toxin is the major mycotoxin that globally contaminates aquafeeds, especially in tropical regions. It is involved in disease and the mortality of aquaculture species [43]. AFB_1_ exposure in fish might result in changes in hematological indices and serum biochemistry of fish [44]. According to previous evidence (e.g., [7, 14, 45–50]), elevated toxin levels may lead to reduced growth performance, histopathological changes in the liver and kidneys, and alterations in hematological and biochemical serum parameters in common carp (*cyprinos carpio*), rainbow trout (*Oncorhynchus mykiss*), rohu (*Labeo rohita*), and silver catfish (*Rhamdia quelen*). It has been shown that rainbow trout is a more susceptible fish species to AFB_1_; susceptibility to infectious diseases and mortality might increase depending on the dietary concentration of the toxin and the duration of exposure [51]. In aquaculture production, considerable research has been performed on the toxicity of AFB_1_ on fish species, including rainbow trout [52], sea bass (*Dicentrarchus labrax*) [53], sea bream (*Sparus murata*) [54], and beluga (*Huso huso*) [55]. The other studied species are juvenile hybrid sturgeon (*A. ruthenus* × *A. Baeri*) [18], Nile tilapia [56–59], rohu (*L. rohita*) [49, 60], and red drum (*Sciaenops ocellatus*) [61]. The remaining species included gibel carp (*Carassius gibelio*) [62, 63], channel catfish (*Ictalurus punctatus*) [64], and juvenile hybrid grouper (*Epinephelus fuscoguttatus* ♀ × *Epinephelus lanceolatus* ♂) [65]. The toxic effects of AFB_1_ on various fish species are summarized in Table 1.

##### 2.1.1.1. Fish Growth Performance

From an economical point of view, feed AF contamination is one of the most crucial worries for aquaculture and feed industries [102] since it might affect the growth performance of fish [19, 103].

Salem et al. [104] found a significant reduction in the growth performance and survival rate of Nile tilapia following dietary exposure to AFB_1_. It has been reported that dietary AFB_1_ decreased the growth rate of the gibel carp by impairing liver function and metabolic disorders [105]. Likewise, Hasanpour et al. [106] concluded that dietary AFB_1_ or ZEN reduced growth indices and affected fish body composition. However, severe changes in fish growth have been noticed in the simultaneous contamination of the diet by both toxins. Barany et al. [107] reported that chronic exposure of sea bream to AFB_1_ impairs growth as well as metabolic and physiological responses of fish to environmental stress, including increased stocking density (i.e., crowding). Conversely, according to Baglodi et al. [108], Indian carp raised on diets containing AFB_1_ at 50, 100, and 150 ppb for 130 days demonstrated no differences in survival, WG, length, or feed conversion ratio (FCR). Meanwhile, Huang et al. [109] and Liu et al. [110] found that AFB_1_ could negatively affect the growth performance and antioxidative capacity of juvenile marbled eel (*Anguilla marmorata*) and growth indices, intestinal health, and muscle quality of hybrid grouper (*E. fuscoguttatus* ♀ × *E. lanceolatus* ♂).

Moreover, it might infiltrate through the blood–brain barrier and affect the brain development of zebrafish embryos by stimulating apoptosis in the brain and axons [34]. In addition, the cytotoxic effects of AFB_1_ in the endothelial cells of the blood–brain barrier were proposed to be connected to silver catfish's behavioral dysfunction [111]. Further, intake of AFB_1_-contaminated food is associated with neurological diseases such as neuropathy, neurological defects, cerebral edema, and even death [112]. Park et al. [113] also concluded that AFB_1_ affected human and zebrafish nervous systems via its antiproliferative and apoptotic properties.

It has been shown that the gastrointestinal tract would be affected by AFB_1_, which might lead to growth deterioration due to mal-nutrition/absorption or endogenous protein loss from increased digestive enzyme synthesis/release [96, 114]. In addition, AFB_1_ might affect intestinal physiology through changes in electrophysiological and morphological properties and mRNA expression of cell-to-cell adhesion proteins. The binding of AFB_1_ to tight junction (TJ) components might result in damage to the intestine and the integrity of the tissue, consequently leading to a leaky gut [114].

##### 2.1.1.2. Digestive Enzyme Activity

Digestive enzymes are essential physiological components of fish growth and development [115]. Their activity also indicates the nutritional status of fish, dietary composition, and digestive tract health [116, 117]. It has been reported that dietary AFB_1_ contamination increases the activity of alkaline protease, lipase, and amylase in common carp [96]. Similar results have been found for various fish species, including rainbow trout [14, 48], tilapia [118], common carp [95], and Chinese sea bass [119] exposed to dietary AFB_1_. However, Fan et al. [120] concluded that feeding on a diet with 50 ppb AFB_1_ decreased the digestive enzyme activity of yellow river carp (*Cyprinus carpio haematopterus*).

##### 2.1.1.3. Immune Indices

Pathogens can breach physical barriers and enter the host, leading to a decrease in immune function and disease resistance [121]. AFB_1_ could affect immune system function in aquatic organisms [122]. Recently, Nazdar et al. [123] reported that AFB_1_ could decrease the survival and functionality of the mouse macrophage RAW264.7 cell line in a dose-dependent manner. Various immune pathways might be inhibited or even stimulated, depending on the concentration of AF to which the animal is exposed or the extent of toxin metabolites produced in the course of toxin biotransformation [124].

Aflatoxicosis leads to the development of deformed cells, eosinophilic cytoplasm, lymphocyte leakage, and cell necrosis in rainbow trout [125]. Further, El-Enbaawy et al. [126] found a decrease in phagocytic activity and neutrophil count in rainbow trout fish [127], as well as a decline in serum immunoglobulin content in *Oreochromis niloticus*. Sepahdari et al. [55] also concluded that feeding beluga with diets containing 100 ppb AFB_1_ remarkably decreased the red blood cell count and blood hemoglobin content of the fish. Moreover, He et al. [92] demonstrated that dietary AFB_1_ decreased the content of antibacterial activity, immunoglobulins, and expression of antimicrobial peptides in the immune organs of grass carp (*C. idella*). Additionally, dietary AFB_1_ affected the expression of various cytokines, including *interleukin* (IL)-6, IL-8, IL-15, interferon-gamma 2, *tumor necrosis factor-α*, IL-17D, and IL-12. Therefore, this AFB_1_ could affect the immunological competence of the skin, the spleen, and the kidney of fish since the spleen and head kidney, along with the skin, are the main body immune organs [55, 92]. In addition, He et al. [92] reported that AFB_1_ decreased the activities of immunological parameters, including lysozyme (LYZ), complement C3 (C3), complement C4 (C4), and immunoglobulin M in grass carp. Similarly, Yang et al. [15] indicated that feeding a diet containing 20 ppb AFB_1_ reduced C3, C4, and immunoglobulin M in juvenile turbot (*S. maximus*).

##### 2.1.1.4. Antioxidant Capacity

Any exposure to AFs might result in increased free radical production/liberation in cells, which could lead to increased tissue malondialdehyde (MDA) content, indicative of increased lipid peroxidation [128]. In other words, lipid peroxidation causes increased tissue MDA content, which might further induce oxidative stress [129, 130]. For instance, Peng et al. [119] found that AFB_1_ increased the MDA content of up to 1.0 ppm in Chinese sea bass. Xue et al. [131] inferred that AFB_1_ induced severe oxidative stress, including increased reactive oxygen species (ROS) and MDA content in gibel carp exposed to 50–100 ppb AFB_1_.

Superoxide dismutase (SOD) and catalase (CAT) are actively involved in decreasing cellular oxidative stress via scavenging ROS [17]. It has been reported that SOD could catalyze the dismutation of superoxide free radicals and thereby alleviate DNA damage. CAT also protects the cell from oxidative injury by catalyzing hydrogen peroxide radicals [132]. Peng et al. [119] concluded that dietary AFB_1_ up to 1.0 ppm resulted in reduced growth, enhanced antioxidant and immune response, decreased intestinal trypsin activity, and impaired intestinal morphology in spotted Leporinus (*Leporinus maculatus*). Further, AFB_1_ has been shown to undesirably affect thyroid gland function and decrease serum T3 and T4 titers in zebrafish larvae. The toxin would also affect the expression of genes involved in oxidative stress and apoptosis [133].

##### 2.1.1.5. Expression of Immune and Inflammatory Genes

Dietary AFB_1_ contamination considerably affects inflammatory and immune responses in different fish species [92, 133, 134]. However, the immune toxicity of the toxin might vary in different fish since they might possess different AFB_1_ biotransformation capabilities [135]. Immune responses and growth performance of fish are interdependent so that any changes in the immune system functionality will finally affect animal growth and body protein accretion. The first immune organ, including the skin, mainly contributes to fish immune responses, where many lymphocytes are naturally present and secrete immunoglobulin and antibacterial compounds. Reduced body protein synthesis might decrease serum antibody content, interfering with proper/suitable lymphocyte functioning and immunological responses. According to the literature, AFB_1_ could adversely affect the structural integrity of highly important supporting organs (the spleen and head kidney) and restrict immunological response in fish [92]. Moreover, the activation of the target of rapamycin (TOR) and nuclear factor kappa B (NF-*κ*B) pathways might be dose-dependently affected by AFB_1_ [136]. It has been reported that any inflammation following the activation of TOR and NF-*κ*B pathways resulted in increased pro-inflammatory cytokine production/liberation and decreased synthesis of anti-inflammatory cytokines [137, 138]. According to Ottinger and Kaattari [139], lymphocytes, monocytes, and neutrophils are responsible for alterations in the expression of LZ, IL-4, and IL-8, so dietary exposure to AFB_1_ might affect their serum content/activity. It has been found that AFB_1_, on the one hand, drastically decreases arginine contents of the spleen and head kidney, which also influences the organ TOR mRNA expression. In addition, the toxin might affect the cell mRNA contents of antibacterial peptides, namely, LAEP-2A, LEAP-2B, hepcidin and *β*-defensin-1, and Mucin-2 immune organs in fish. AFB_1_ also influences the expression of IL-6, IL-8, IL-15, interferon-gamma 2, *tumor necrosis factor*-*α*, IL-17D, and IL-12p40 cytokines [92]. Recently, Ghafarifarsani, Kachuei, and Imani [48] have demonstrated that the expression of IL1-*β*, INF-*γ*, and TNF-*α* genes was increased in rainbow trout fed a diet containing 25 ppb AFB_1_.

##### 2.1.1.6. The Liver Tissue Injury and Expression of Hepatic Antioxidant Enzymes

The liver is the main organ that is responsive to absorbed AFB_1_ [5, 7, 140], and hepatic enzymes are considered indicators of cellular damage and tissue function impairment [141, 142].

The liver is involved in metabolizing different xenobiotics, including toxins, and might be affected by aflatoxicosis [124, 143]. Through blood circulation, AFB_1_ is immediately transferred to the liver and metabolized by hepatocytes. Cytochrome P450 (CYP450) enzymes metabolize AFB_1_ to AFB_1_-exo-8,9-epoxide, a highly toxic and reactive AFB_1_ metabolite that can react with different biomolecules, including DNA, RNA, and protein. It could also finally inactivate the p53 gene. The event might eventually result in GC to TA mutagenesis [10]. AFB_1_-DNA conjugate was reported in the liver of AFB_1_-exposed rainbow trout and Atlantic salmon. Naturally, a higher half-life of AFB_1_-DNA in fish hepatocytes compared to mammals might imply that its enzymatic removal is insufficient in fish, indicating a higher probability of mutation in fish [144].

The living cells contain antioxidant enzymes (e.g., SOD, CAT, glutathione peroxidase, and glutathione reductase), for protection against oxidative stress due to xenobiotic metabolism and/or resultant ROS. The immune system of zebrafish (*Danio rerio*) was responsive to oxidative damage following excess ROS production via NF-E2-related factor 2 [145]. AFB_1_ damages the hepatocyte cell membrane and results in serum liver enzyme leakage. Those enzymes activity in serum samples were used as the biological markers of liver tissue damage in common carp and northern snakehead (*Channa argus*) [146, 147]. Recently, Di Paola et al. [133] investigated the effect of AFB_1_ on Zebrafish embryos and found that AFB_1_ increased oxidative stress indices, including activity of SOD, CAT, GST, and CYP450, along with tissue MDA and apoptotic protein contents. Disturbed cellular oxidation-reduction status and tissue damage following oxidative stress were reported in the liver of Chinese sea bass [119] and Stellate sturgeon (*A. stellatus*) fingerlings [90]. Oxidative stress increased hepatic lipid peroxidation and tissue ROS production in common carp. Lipid transportation was adversely affected following hepatic AFB_1_ bioaccumulation [148]. Indeed, increased liver lipid deposition was reported in red drum [61] and juvenile rainbow trout [46] exposed to AFB_1_. Impaired hepatic lipid metabolism, lipid peroxidation, or lipoprotein synthesis following AFB_1_ exposure was also reported in gibel carp [62]. Furthermore, hepatic cell damage by AFB_1_ exposure resulted in decreased whole-body protein and lipid contents in hybrid striped bass (*Morone chrysops × M. saxatilis*) [124]. It has also been shown that any liver damage caused by oxidative stress leads to increased tissue protein degradation and reduced protein synthesis in animals [46, 149].

##### 2.1.1.7. Intestinal Tissue Structure and Barrier Proteins

The intestinal epithelial integrity by adhesion junctions, TJs, and desmosomes plays a vital role in intestinal permeability, nutrient uptake, toxins uptake, bacterial translocation, and immune response, recently known as the main gut health components. TJs, as the major functioning components of the intestinal barrier, seal the intercellular spaces of epithelial cells [150, 151]. It has been shown that intestinal integrity through clathrin-mediated endocytosis was affected by AFB_1_ [151].

Huang et al. [152] concluded that feeding an AFB_1_-contaminated diet imposed intestinal oxidative damage, TJ destruction, and epithelial cell apoptosis, which could adversely affect the integrity of the intestine in juvenile grass carp. In addition, claudin and occludin and their interaction with signaling molecules regulate the permeability of junctions in the gastrointestinal tract. In sea bream (*S. aurata*), AFB_1_ affected claudin proteins in intestinal TJs and resulted in cell necrosis with mononuclear cell penetration [114]. Feeding rainbow trout with an AFB_1_ contaminated diet caused infiltration of inflammatory cells into the underlying intestinal mucosal layer [82]. Moreover, the expression of caspase-3, a central effector of cell apoptosis, increased in goldfish (*Carassius auratus*) [63] and in common carp [95] following AFB_1_ exposure.

The digestive tract is the main route of feed-born toxins' entry to the body [153], so that any dietary exposure to AFB_1_ might affect fish susceptibility to secondary infectious microorganisms [154]. For instance, gastrointestinal microbiota was affected in turbot feed AFB_1_-contaminated diet [15]. Further, the structural disruption of intestinal epithelial cells leads to increased feed-born toxins or antigen uptake into blood circulation with subsequent susceptibility to pathogens [150, 155].

AFB_1_ increased the expression of IL-1*β* and TNF-*α* mRNA in rainbow trout, which resulted in intestinal inflammation, severe tissue damage, and reduced nutrient bioavailability [48]. Changes in intestinal villus morphology and damaged enterocytes were observed following dietary AFB_1_ exposure in juvenile gilthead seabream [114], Chinese sea bass [119], and rainbow trout [156]. Furthermore, Zhang et al. [84] reported considerable changes in the abundance of intestinal bacteria in turbots fed a diet containing AFB_1_ in comparison to the control group.

##### 2.1.1.8. Gill Tissue Damage and Disturbed Lamellar Ventilation

Pathological changes in gill tissue might be indicative of exposure to toxins or xenobiotics [63, 157]. For instance, AFB_1_ adversely altered the structural barrier of gills and remarkably lowered TJ proteins and anti-inflammatory gene expression in grass carp [158]. The gill lamellae hypertrophy, increased secondary lamella thickness, and increased mucus secretion were found in rohu exposed to AFB_1_ [76]. It has been recently reported that any dietary exposure to AFB_1_ resulted in pathological changes in goldfish gills [63]. Similarly, lamellae edema and epithelial necrosis with physiological consequences were confirmed in Nile tilapia [159]. Cell necrosis and lamellae hemorrhage in major Indian carp, rohu, were detected following aflatoxicosis [160]. In addition, gill hyperplasia and epithelial disruption were observed in Stellate sturgeon (*A. stellatus*) [89], rainbow trout (*O. mykiss*) [82, 161], and rohu (*L. rohita*) [76].

The exfoliation of epithelial cells in lamellae could lead to an increased distance between oxygen-containing water flow and blood circulation and an insufficient supply of oxygen, and consequently, severe secondary lamellae necrosis, which was a principal limiting factor for metabolite excretion via gills [161, 162].

## 3. Worldwide AFB_1_ Occurrence in Aquafeed and Lowest Observable Effect Levels (LOELs)

In spite of national and international constant *surveillance* to limit/manage fungal toxins, it has been reported that approximately 475 million tons of feedstuffs and forages have been consumed only in the EU. While the mycotoxin contents of the feedstuffs are well below the accepted maximum levels for animals, their co-occurrence is now a worldwide feed supply chain concern [27, 163, 164]. Generally, AFB_1_ is more prevalent in tropical regions due to warm, humid conditions [165]. For instance, AFB_1_ content of fish feed in Asia and Africa typically ranges from 51.83 μg/kg (51.83 ppb) to 220.61 μg/kg (220.61 ppb), while in the EU region, it is 0.43 μg/kg (0.43 ppb) on average [88, 166, 167].

As discussed earlier in the present review, some aquatic species, including channel catfish, Coho salmon, and tilapia, are less susceptible to aflatoxicosis thanks to their higher metabolic capacity to biotransform AFs [168]. According to the International Agency for Research on Cancer, AFs are the primary cause of human carcinoma [169]. It is highly recommended that allowable ranges of feed/food (ingredients) toxins should be established as a safe standard rate with a safety margin. Generally, the acceptable value for Nile tilapia is <100 ppb. The legal limit of AFs in feed for all animal species is 50 ppb in Brazil. In the United States and EU, however, the safe level is 10 ppb for some agricultural products/commodities and livestock products, respectively. The acceptable daily intake of 5 ppb AFB_1_ is introduced by the Food and Drug Administration. However, the maximum authorized concentration of 20 ppb is also established by the United States Food and Drug Administration [170] for total AFs (AFB_1_ mixed with AFB_2_, AFG_1_, and AFG_2_). However, the EU has defined maximum limits of 5 and 12 ppb for AFs in feed and foods, respectively [14].

Raghavan et al. [18] found that juvenile hybrid sturgeons (*A. ruthenus* × *A. baeri*) are sensitive to dietary AFB_1_ contents of >10 μg/kg feed (10 ppb). AFB_1_ content of commercial fish feed was demonstrated to be less than 10 μg/kg (10 ppb) [171, 172]. However, higher dietary contents were also unavoidable [173]. Meanwhile, Pietsch [134] considered that 4.30 μg/kg feed (4.30 ppb) might be a safe AFB_1_ contamination threshold in commercial feeds. Similarly, Nácher-Mestre et al. [174] concluded that the overall level of mycotoxin in fish feed was below the maximum residue limit suggested by Commission Recommendation 2006/576/EC, and no mycotoxin transfer might occur from feeds to fish fillets.

Furthermore, the lowest and highest threshold concentrations are 1.69 and 8.70 ppb, respectively, and with an average concentration of 4.30 ppb, 5% of the fish population might be at risk of aflatoxicosis. Early signs of body composition changes and oxidative stress following dietary AFB_1_ exposure would be observable at 563 ± 252 and 1598 ± 1467 ppb, respectively [30]. LOEL is different for various fish species. For instance, exposure to 20–200 ppb AFB_1_ did not affect common carp [175, 176]. However, juvenile common carp showed decreased growth indices following dietary exposure to 100 ppb AFB_1_ [177]. Meanwhile, exposing common carp to 2 ppb AFB_1_ resulted in liver injury and histopathological alterations at 20–200 ppb AFB_1_. However, the lowest LOEL for genotoxicity and immunosuppression were 317 ± 136 and 1770 ± 630 ppb in different fish species, respectively [30]. He et al. [92] also discussed that LOEL for the normal functioning of the immune organs (the skin, spleen, and head kidney) would be 29.48 ppb AFB_1_ in grass carp. According to Alinezhad et al. [46], the early signs of reduced growth indices would be observed following dietary exposure to >5 ppb AFB_1_ in rainbow trout. As discussed above, there are considerable reports regarding harmful and lethal concentrations of AFB_1_ in aquafeed. However, acceptable thresholds of AFB_1_ in different fish species require further studies [48, 50, 135].

## 4. Conclusion

AFB_1_ is considered a potential threat to the aquaculture industry regarding aquatics and consumer health. Dietary AFB_1_ contamination influences the immune system and growth performance, resulting in economic worries and decreased farm profitability. Therefore, it is highly recommended that safety thresholds or standards be determined for feed and final products of AFB_1_ contents. To introduce such standards, various factors, including fish species, developmental stage, culture condition, and facilities, along with final product safety, should be taken into consideration. Studies on how to manage/control the occurrence of toxins in aquafeed must also be conducted as well. Considering that there is high variation in feed (stuff) mycotoxin content from one place to another, close collaboration between scientists and legislation authorities is required regarding sampling methods/frequencies, processing, and/or analyses. In addition, developing quick and handy methods of detecting (multi)-mycotoxin contamination is necessary.

## Figures and Tables

**Table 1:** Effects of dietary AFB_1_ contamination on different fish species.

Species	Dose	Effect	Reference
Nile tilapia (*Oreochromis niloticus*)	100 ppb 200 ppb	Growth impaired Mortality	El-Banna et al. [56]
	2000 or 4000 ppb	Reduced weight gain and decreased body lipid content	Hussain, Manteen, and Gatlin [5]
	200 ppb	Decreased total erythrocyte and leucocyte count, serum liver enzymes leakage, hemoglobin count decreased; reduced weight gain	Selim, El-hofy, and Khalil [44]
	375, 752, 940, 1500, 1880, 3000 ppb	Decreased FI and growth rate, liver atrophy	Chávez-Sánchez, Martinez-Palacios, and Osorio-Moreno [57]
	100,000 ppb	Lipofuscin and irregularly hepatocellular nuclei, weight loss, severe hepatic necrosis, and mortality	Tuan et al. [58]
	100 ppb	Increased liver enzymes, reduced growth rate, and weight gain	Mahfouz and Sherif [66]
	100 ppb	Weight loss, changes in blood parameters, and liver necrosis	Abdelhamid et al. [67]
	3000 ppb	Lower SGR	Shehata, El-Melegy, and Ebrahim [68]
	200, 250 ppb	Mortality	Naiel, Ismael, and Shehata [69]
	100 ppb	Severe liver tissue vacuolation and lipid accumulation	Kenawy et al. [70]
	100 ppb	Decreased growth	Encarnacao et al. [71]
	150 ppb	Serious toxic impacts and negative effects on health performance	Mehrim and Salem [72]; Zychowski et al. [73]

Tetrahybrid red tilapia	<5 ppb	Pale gills, liver damage, poor growth rates, and immune suppression	Conroy [74]

Rohu (*L. rohita*)	1250 ppb	Reduction in nonspecific immunity	Sahoo and Mukherjee [75]
	7500, 1125 ppb 1250, 2500 ppb 12,000, 13,300 ppb	Acute toxicity Subchronic toxicity Necrosis of gill lamellae, primary lamellar hyperplasia	Sahoo and Mukerjee [76]
	10, 20 ppb 40 ppb	Liver tissue mild edema Swollen hepatocytes, kidney mild hemorrhages, decrease total erythrocyte, and hemoglobin count	Mohapatra et al. [77]
	25, 50 ppb 100 ppb	Reduced growth performance, growth depression Reduced growth indices and survival rate	Bhatt et al. [49]
	100 ppb	Cytoplasmic vacuolization in hepatocytes and hepatic tissue showing loss of membrane integrity, along with diffused hepatocytes and hyperplasia	Bhatt et al. [78]
	1250 ppb	Increased serum lysozyme activity, enhanced phagocytic ratio, and immunostimulatory effects	Sahoo and Mukherjee [76]

Seabass (*D. labrax*)	180 ppb 18 ppb 4.25 ± 0.85 ppb	Abnormal behavioral Increased ALT, AST, and ALP enzymes, decrease in plasma proteins Serious health problems in exposed fish and a high risk to fish consumers	El-Sayed and Khalil [79]

Rainbow trout (*O. mykiss*)	500 ppb	Acute toxicities	Lovell [80]
	810 ppb	Acute toxicities	Bauer, Li, and Sinnuhuber [81]
	25 ppb≥ 50 ppb≤	Persisting inflammatory response without mortality Decreased LYZ, TP, and ALB and increased inflammatory cytokines Villi destruction and necrosis, hyperplasia, and edema of gill lamellae	Ghafarifarsani et al. [50]
	25 ppb≥ 50 ppb≤ 50 ppb	Infiltration of inflammatory cells into the underlying layers, necrosis, hyperplasia, atrophy, and severe destruction of gills Liver tissue damage and hepatocyte changes	Imani et al. [82]
	50 ppb	Destruction of intestinal villi	Mahmoudi et al. [83]

Juvenile turbot (*Scophthalmus maximus* L.)	100 ppb	Negatively affected liver catalase activity and intestinal microbiota	Zhang et al. [84]

Silver catfish, Jundia (*R. quelen*)	204 ppb 350 ppb	Lower weight and length gain Alterations in the liver and tissues	Lopes et al. [85]

Channel catfish (*I. punctatus*)	10,000 ppb	Decreased growth performance, anemia, and liver and gastric necrosis	Jantrarotai and Lovell [64]
	12,000 ppb	Regurgitating stomach contents, pale organs of moribund fish, istological lesions, and mortality	Jantrarotai, Lovell, and Grizzle [86]

Yellow catfish (*Pelteobagrus fulvidraco*)	200 ppb<	Growth performance (WG, SGR), lower survival rate, and increased FCR	Wang et al. [87]

Tra catfish (*Pangasius hypophthalmus*)	500, 1000 ppb 50, 100, 250 ppb	Increased HIS AST, ALT, and liver damage	Gonçalves et al. [88]

White surgeon (*H. huso*)	75, 100 ppb	Altered feed conversion and weight gain and decreases in growth	Sepahdari et al. [55]

Juvenile hybrid sturgeon (*A. ruthenus ♂* × *A. baeri ♀*)	40 ppb 80 ppb	Mortality High mortality, decreased hematocrit value, nuclear hypertrophy, and hyperchromasia	Raghavan et al. [18]

Stellate sturgeon (*Acipenser stellatus*)	1500 ppb 3500 ppb	8% mortality 50% mortality	Santacroce et al. [7]
	75, 100 ppb	Bleeding points in the gills and head, hyperplasia and destruction of the epithelial tissue of the gills lamellae, and necrosis of the liver cell	Motallebi Moghanjouei [89]
	1500, 1850, 2300, 2850, 3500 ppb	Increased liver enzymes (AST, ALT, and ALP) and mortality rate	Jalilpour et al. [90]

Grass carp (*Ctenopharyngodon idella*)	147 ppb	Suppressed Nrf2 signaling—a decrease in growth and antioxidant enzymes—and disruption of the integrity and TJ protein	Zeng et al. [91]
	85.94 ppb<	Decreased expression of genes *β*-defensin-1, LEAP-2A, Mucin2, and LEAP-2B	He et al. [92]

Gibel crap (*C. gibelio*)	5 ppb	Reduced growth	Han et al. [45]
	2000 ppb<	Fecundity is reduced and tissue accumulation	Huang et al. [93]

Common carp (*C. carpio*)	100,000 ppb	Immunosuppression	Sahoo and Mukherjee [94]
	200, 400 ppb	No mortality	Al-Rubaiy et al. [95]; Tasa et al. [96]
	500, 1000, 2000 ppb	Decreased weight gain, histopathological changes	Rhadi, Rudainy, and Attee [97]

Salmon (*Oncorhynchus kisutch*)	10,000 ppb	Acute toxicities	Schoental [98]

Mosquitofish (*Gambusia affinis*)	4640 ppb	Acute toxicities and mortality	McKean et al. [99]

Tambaqui fingerlings (*Colossoma macropomum*)	500 ppb<	Decreases in the WG, FI, and FE	Nunes et al. [100]

Lambari fish (*Astyanax altiparanae*)	10 ppb	Accumulation in fish liver and muscle	Michelin et al. [101]

Abbreviations: ALB, albumin; ALP, alkaline phosphatase; ALT, alanine transaminase; AST, aspartate aminotransferase; FCR, feed conversion ratio; FE, feed efficiency; FI, feed intake; HIS, hepatosomatic index; LYZ, lysozyme; Nrf2, NF-E2-related factor 2; SGR, specific growth rate; TJ, tight junction; TP, total protein; WG, weight gain.

## Data Availability

Data sharing is not applicable to this article, as no new data were created or analyzed in this study.
